# Magnesium and nerve injury: Mechanisms and applications

**DOI:** 10.4103/NRR.NRR-D-25-00263

**Published:** 2025-08-13

**Authors:** Hongye Yan, Su Pan, Longchuan Zhu, Weijian Kong, Zhiping Qi

**Affiliations:** 1 Department of Orthopedic Surgery, the Second Hospital of Jilin University, Changchun, Jilin Province, China; 2 Department of Nuclear Medicine, the Second Hospital of Jilin University, Changchun, Jilin Province, China

**Keywords:** brain injury, magnesium ions, magnesium-based materials, mechanism of nerve injury, nerve injury repair, nerve regeneration, optic nerve injury, peripheral nerve injury, spinal cord injury

## Abstract

Magnesium is a vital mineral that plays an important role in recovery from nerve injury recovery by inhibiting excitotoxicity, suppressing inflammatory effects, reducing oxidative stress, and protecting mitochondria. The role of magnesium ions in the field of nerve injury repair has garnered substantial attention. This paper aims to review the mechanisms of action and potential applications of magnesium in nerve injury repair. Magnesium ions, as key neuroregulatory factors, substantially alleviate secondary damage after nerve injury by inhibiting N-methyl-D-aspartate receptors, regulating calcium ion balance, providing anti-inflammatory and antioxidant effects, and protecting mitochondrial function. Magnesium ions have been shown to reduce neuronal death caused by excitotoxicity, inhibit the release of inflammatory factors, and improve mitochondrial function. Additionally, magnesium materials, such as metallic magnesium, magnesium alloys, surface-modified magnesium materials, and magnesium-based metallic glass, exhibit unique advantages in nerve repair. For example, magnesium materials can control the release of magnesium ions, thereby promoting axonal regeneration and providing mechanism support. However, the rapid corrosion of magnesium materials and the limited amount of research on these materials hinder their widespread application. Existing small-sample clinical studies have indicated that magnesium formulations show some efficacy in conditions such as migraines, Alzheimer’s disease, and traumatic brain injury, offering a new perspective for the application of magnesium in nerve injury rehabilitation. Magnesium ions and their derived materials collectively hold great promise for applications in nerve injury repair. Future efforts should focus on in-depth research on the mechanisms of action of magnesium ions and the development of magnesium-based biomaterials with enhanced performance. Additionally, large-scale clinical trials should be conducted to validate their safety and efficacy.

## Introduction

Spinal cord injury (SCI) and peripheral nerve injury (PNI) profoundly affect patients’ daily activities (Song et al., 2025; Yan et al., 2025). SCI can be categorized into traumatic and non-traumatic SCI on the basis of the cause of the injury (Liang et al., 2025). This paper mostly focuses on the ramifications of traumatic SCI. Traumatic SCI may result from vehicular collisions, falls from elevations, spine fractures, or pathogenic injuries. In patients with SCI, the pathway for transmitting information from the brain to the body may be disrupted or severed, resulting in a total loss of sensation, movement, and reflexes, as well as complete (or partial) loss of sphincter function and potential paralysis. These effects greatly diminish the quality of life of patients and cause considerable distress to them and their families (Hu et al., 2023). An epidemiological study has indicated that the annual incidence of SCI is 40–80 cases per 1 million individuals (Kumar et al., 2018), and the annual cost of caring for patients with SCI is US$10 billion (Thompson et al., 2015). Currently, patients with SCI worldwide are typically aged between 16 and 30 years (Ding et al., 2022). Furthermore, PNI has been categorized into five degrees (Nocera and Jacob, 2020): Grade I is neurapraxia, which is characterized by intact axons and diminished nerve conduction velocity. Grade II is marked by axonal rupture with an intact perineurium and endoneurium. Grade III indicates compromised axons, myelin sheaths, and endoneurium, with an intact and normal perineurium. Grade IV involves obliteration of the nerve bundle, axon, endoneurium, and perineurium, while the epineurium remains unscathed, preserving the continuity of the nerve trunk. In Grade V, both the nerve bundle and the epineurium are torn, and the nerve trunk is entirely compromised and lacks continuity. This discussion mostly focuses on the ramifications of fifth-degree injuries. Complete rupture of peripheral nerves is frequently induced by trauma or compression. After nerve injury, atypical communication between the brain and the affected organs and muscles results in motor and sensory function impairment or potential disability in the region innervated by the compromised nerve (Prakash and Udupi Bidkar, 2016). Epidemiological research indicates that the annual prevalence of peripheral nerve damage is approximately 13–23 cases per 100,000 individuals globally, with upper limb nerve injuries being more prevalent. The incidence rate significantly differs across various regions. In several developing nations, the prevalence of PNI is notably elevated due to the significant occurrence of traffic accidents and workplace injuries. Rehabilitation of PNI is prolonged and expensive, imposing a substantial financial burden on patients and their families and resulting in productivity losses (Taylor et al., 2008).

From a pathophysiology standpoint, SCI encompasses a sequence of cascading events at the injury site following the main damage, which includes bleeding, ischemia, oxidative stress, inflammatory response, and neuronal cell death (Hu et al., 2023). The primary signs of PNI are Wallerian degeneration and regeneration at the injury site (Li et al., 2023).

The existing therapeutic modalities for SCI include pharmacological treatment, cellular therapy, electrical stimulation, and biomaterials-based tissue engineering therapy (Hu et al., 2023). Although these therapeutic modalities can facilitate SCI repair through neuroprotective, anti-inflammatory, and antioxidant actions, their long-term efficacy in spinal cord regeneration is limited. Spinal cord damage is frequently followed by fibrous and glial scars, which exert an inhibitory influence on axonal regrowth (Brockie et al., 2024). Tissue engineering therapy offers topological, biophysical, and biochemical cues to modulate intrinsic neuronal regeneration and enhance the external milieu for SCI healing (Shen et al., 2022). Consequently, tissue engineering therapies associated with biomaterials possess extensive potential. The restoration of PNI encompasses numerous elements, including Schwann cells, fibroblasts, extracellular matrix, injured distal nerve stumps, inflammatory cytokines, and neurotrophic factors (Wang et al., 2020). The primary existing therapeutic modalities for PNI are microsurgery, endogenous nerve grafting, and tissue engineering techniques (Hu et al., 2023). Although autologous transplantation of organs is the gold standard for treating PNI, the scarcity of donors restricts its implementation. Conversely, the problems associated with long nerve gaps also restrict the use of autologous transplantation. Tissue engineering therapy has recently gained acceptance for repair of peripheral nerve damage. For example, tissue engineering technology that combines seed cells, neurotrophic factors, and nerve guide catheters offers great potential in the treatment of long-gap nerve injuries (Li et al., 2021).

The history of the use of magnesium commenced in 1755 when Scottish physician and scientist Joseph Black first identified it as an element. In 1892, Austrian physician Erwin Payr introduced many clinical applications and documented advancements in biodegradable magnesium implants (Witte, 2010). With progress in biomaterial research, magnesium-based materials began to be used in neural healing, with studies indicating their potential to enhance nerve regeneration (Vennemeyer et al., 2014; Kim et al., 2025a, b). Conversely, research on magnesium-based materials has shown that magnesium ions are the primary agents in brain healing. Magnesium ions participate in the synthesis of membrane phospholipids, signal transduction (Stangherlin and O’Neill, 2018), and myelin and synapse development (Sun et al., 2016), and they govern the transmission of neurotransmitters, including dopamine and serotonin (5-HT). Simultaneously, magnesium ions can facilitate axon growth and neural stem cell proliferation, modulate inflammatory responses, mitigate oxidative stress-induced damage, and suppress cellular death. Furthermore, Mg^2+^ ions constitute the primary divalent metal cations within cells and are vital trace elements in the organism. Simultaneously, magnesium ions also serve as cofactors in numerous enzymatic processes, particularly those involving nucleotides as cofactors or substrates. Magnesium ions are essential for protein synthesis, nucleic acid synthesis, the cell cycle, cytoskeletal integrity, mitochondrial integrity, and the binding of molecules to the plasma membrane (Saris et al., 2000).

This review delineates the mechanisms underlying SCI, PNI, brain injury, and optic nerve injury, alongside the utilization of magnesium ions and magnesium-based materials in the restoration of nerve injuries. The following sections outline the historical evolution of the use of magnesium in this clinical context, along with its safety and the current status of its clinical application. The objective of this review is to encapsulate the correlation between magnesium and nerve injury while proposing innovative concepts for utilizing magnesium-based materials in future nerve injury restoration.

## Search Strategy

The Web of Science Core Collection was designated as the database for data extraction. We searched for pertinent papers using the following strategy: TS=(nervous system injury) and TS=(magnesium). The search was restricted to “article” or “review” types, studies published in English, and publication dates between January 1, 2000, and December 31, 2024. A total of 1578 unique English documents were acquired, including 1170 articles and 408 reviews.

## Role of Magnesium in the Nervous System

### Physiological roles of magnesium

Magnesium is a divalent cation that is vital to human health and performs important functions in cells. It has been shown to substantially improve human health and is essential for many physiological processes. Numerous biological processes, such as oxidative phosphorylation, energy production, glycolysis, and the creation of proteins and nucleic acids, depend on magnesium ions (Santos et al., 2023; Pethő et al., 2024). In addition to the functions mentioned above, magnesium ions also play vital roles in cell signaling, ion transport across cell membranes, muscle contraction, mitochondrial function, and regulation of neuronal excitability. As a cofactor in over 600 enzyme events, magnesium ions are essential for a wide range of physiological processes and notably influence general health and well-being (Fatima et al., 2024).

### Magnesium and the stability of neuronal cell membranes

Ion pumps that are located on the cell membrane are known to be intimately connected to the homeostasis of cells. Magnesium ions are cofactors for a wide variety of enzymes, as described earlier. Therefore, magnesium ions have the ability to indirectly maintain the stability of the cell membrane by supporting the functioning of the sodium-potassium pump. This helps maintain the stability of the ion gradient both inside and outside the cell, which is essential for the generation of both resting and action potentials. Magnesium is also involved in the creation of the phospholipid bilayer that constitutes the cell membrane, and it plays a specific role in the process of ensuring that the cell membrane retains its structural integrity (Zhang et al., 2021a).

### Role of magnesium in neurotransmitter release and synaptic transmission

Signal transduction among some cells occurs through glutamate and calcium ions. In typical conditions, when the synapse receives the appropriate signal, the receptors on the cell membrane are engaged. This allows the entry of calcium ions, which triggers the release of neurotransmitters from the vesicles and thereby facilitates signal transmission between cells. Magnesium ions facilitate the preservation of vesicle membrane fluidity and ensure the timely release of neurotransmitters from the vesicles. Furthermore, an increase in magnesium ion concentration between synapses can antagonize calcium ions to some degree, allowing timely termination of neurotransmitter release and preventing excitotoxicity. In some instances, insufficient magnesium ion concentration may inhibit the release of neurotransmitters, causing synaptic dysfunction. Consequently, magnesium ions are crucial for properly controlling neurotransmitter release and synaptic transmission (Kumar et al., 2024; Stanojevic et al., 2025).

## Mechanisms of Nervous System Damage

### Spinal cord injury

SCI is a multifaceted pathological process consisting of primary and subsequent injuries (Sousa et al., 2025). The former pertains to the acute phase following injury and primarily involves traumatic damage to the spinal cord; the latter denotes a cascade of molecular processes at the injury site that result from functional impairments after the primary injury (Hu et al., 2023b), encompassing excitotoxicity induced by elevated Ca^2+^ levels (Baklaushev et al., 2019), mitochondrial impairment (Schmidt and Quintá, 2023), oxidative stress (Chio et al., 2022), and inflammatory response (Hellenbrand et al., 2021).

**Figure 1:**
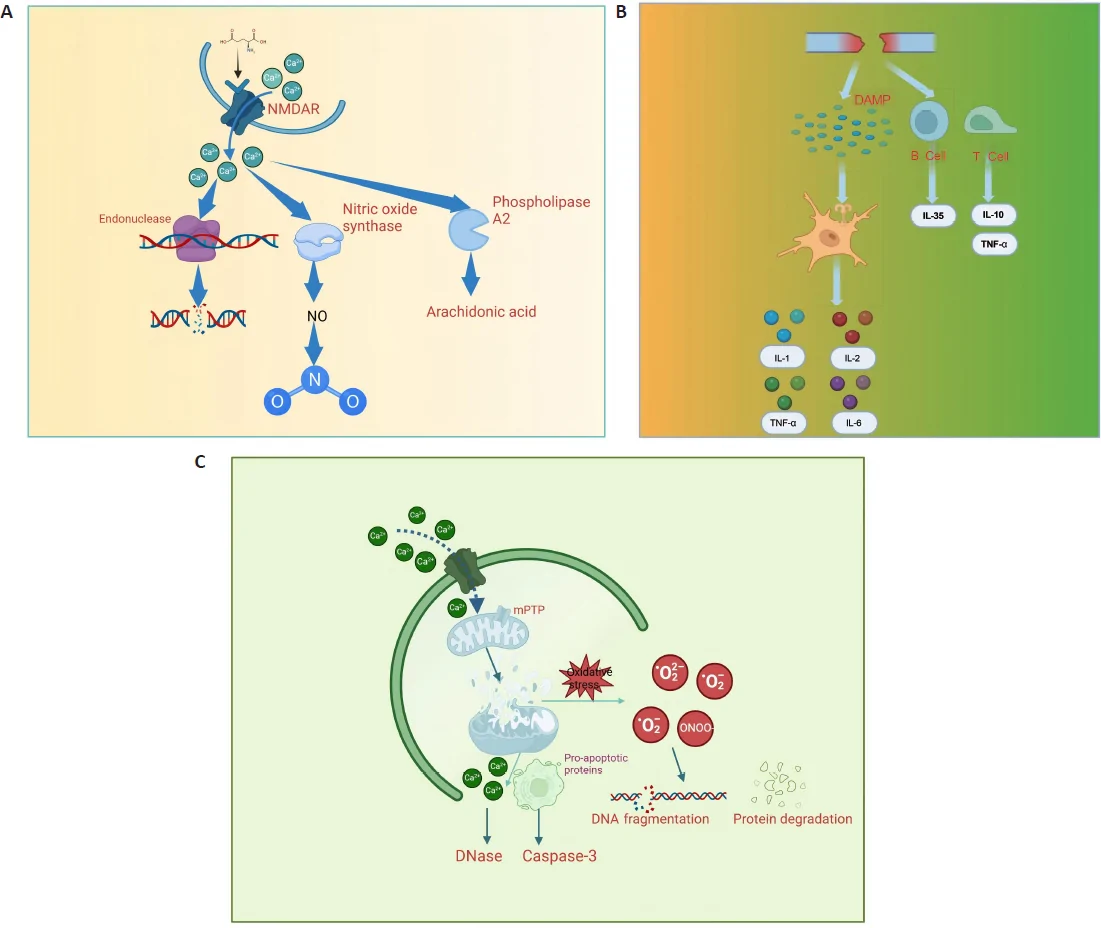
Pathophysiological mechanism of SCI. (A) A significant influx of Ca^2+^ occurs following SCI, which increases the permeability of the cell membrane to Ca^2+^. This accumulation of Ca^2+^ leads to the large-scale release of glutamate. Excessive Ca^2+^ activates endonucleases, resulting in DNA breakdown, stimulates nitric oxide synthase to produce NO, and activates phospholipase A2 to generate arachidonic acid, ultimately causing neuronal cell death. (B) Inflammatory response in neural injury: DAMPs are released due to SCI, which can bind to toll-like receptors on microglia. This activation prompts microglia to release innate pro-inflammatory cytokines such as TNF-α, IL-1, IL-2, and IL-6. Additionally, lymphocytes exacerbate the damage by producing pro-inflammatory cytokines and forming various cell subpopulations. (C) Mitochondrial impairment and oxidative stress in neural damage: Following SCI, a significant amount of Ca^2+^ enters the mitochondria, ultimately leading to mitochondrial rupture. The release of high levels of Ca^2+^ and pro-apoptotic proteins into the cytoplasm activates the downstream apoptotic pathway mediated by DNase and Caspase-3 in the nucleus, resulting in the hydrolysis of cytoskeletal proteins. Furthermore, reactive oxygen species generated from mitochondrial damage can bind to proteins and DNA, causing their degradation and breakdown. Created with BioRender.com. DAMP: Damage-associated molecular patterns; IL: interleukin; mPTP: mitochondrial permeability transition pore; NMDAR: N-methyl-D-aspartate receptors; NO: nitric oxide; NOS: nitric oxide synthase; ONOOCO_2_^–^: peroxynitrite anions; SCI: spinal cord injury; TNF-α: tumor necrosis factor alpha.

Glutamic acid is an excitatory amino acid within the central nervous system. It can bind to and interact with the organism’s N-methyl-D-aspartate receptors (NMDARs). It is typically deposited in neurons next to axon terminals. Vesicles are released into the synapse upon Ca^2+^ influx (Katayama et al., 1990; Verma et al., 2022). Spinal cord damage can result in significant hemorrhage in the gray matter, causing hemorrhagic necrosis and ensuing central myelomalacia (Tator and Koyanagi, 1997; Hernandez-Gerez et al., 2019). Furthermore, the disruption of blood flow and the presence of hypotension/hypoperfusion in the affected region can cause elevated cell membrane permeability, significantly enhancing the influx of Ca^2+^ and resulting in substantial internalization of Ca^2+^ flow, resulting in a significant buildup of intracellular Ca^2+^. The substantial concentration of Ca^2+^ induces a significant release of glutamate, resulting in excitotoxic neuronal death. Excess Ca^2+^ levels activate endonucleases to cause DNA degradation; activate proteases to induce cytoskeletal breakage; activate NO synthase to produce NO and subsequently nitrite; and activate phospholipase A2 to induce arachidonic acid synthesis. These processes eventually result in reduced intracellular adenosine triphosphate levels and decreased pH, and ultimately cause the demise of neuronal cells (Lau and Tymianski, 2010; **Figure 1A**).

Mitochondria are the cell powerhouses that produce ATP for metabolism through oxidative phosphorylation in the electron transport chain (ETC). The mitochondrial permeability transition pore (mPTP) is a key protein ion channel that spans the inner and outer membranes of mitochondria, playing an important role in maintaining ionic balance within these organelles (Cao et al., 2013). In SCI, the permeability of both cell and mitochondrial membranes to Ca^2+^ are increased, resulting in enhanced calcium levels inside the mitochondria. Ca^2+^ elevation activates several proteases and lipolytic enzymes in mitochondria that cause mitochondrial membrane damage, resulting in a decrease in ATP levels. In addition, the mPTP will remain open once the levels of Ca^2+^ exceed a certain threshold. As such, the inner mitochondrial membrane potential is lost, resulting in cessation of ATP synthesis. Water molecules entering the mitochondria rush to fill up the matrix and swell it to exert excessive force on the outer membrane. Therefore, large amounts of Ca^2+^ and pro-apoptotic proteins are released in cytoplasm, which induces downstream apoptotic pathways controlled by DNase (caspase-3) that cause degradation of cystoskeletal proteins (Golpich et al., 2017).

The presence of an inflammatory response in SCI is significant. SCI initially results in compromise of the blood–spinal cord barrier and vascular endothelial cells, triggering the production of damage-associated molecular patterns (DAMPs) that can be recognized and bound by toll-like receptors on microglia (Zhang et al., 2025). Subsequently, microglia are activated and secrete innate proinflammatory cytokines, including tumor necrosis factor (TNF)-α, interleukin (IL)-1, IL-2, and IL-6 (Nesic et al., 2001; He et al., 2022). These inflammatory factors exacerbate vascular permeability, neuronal apoptosis, peroxidation of lipids, DNA fragmentation, and obstruction of the mitochondrial respiratory chain. Furthermore, neutrophils are activated at the onset of spinal cord damage, generating reactive oxygen species (ROS) and nitrosyl free radicals to sanitize the affected region; nevertheless, their disinfecting capacity is frequently unregulated, causing oxidative stress (Trivedi et al., 2006; Freyermuth-Trujillo et al., 2022). Lymphocytes are implicated in the injury during the second stage. Following SCI, lymphocytes are drawn to the injury site by the microenvironment and molecular signals, resulting in the formation of cell subsets and the production of proinflammatory cytokines such as interferon-gamma and transforming growth factor beta, which subsequently hinder functional recovery at the injury site and worsen injury-induced pathology. The literature indicates that the role of the inflammatory response following spinal cord damage is highly contentious. Although inflammation is predominantly recognized as neurotoxic, substantial data indicates that it also possesses neuroprotective properties (Jones et al., 2004; Ankeny and Popovich, 2007). Consequently, the involvement of inflammation in SCI appears to be dual-faceted, with no clear, straightforward method to differentiate between the two separate components. This process is depicted in **Figure 1B**.

The mechanisms underlying secondary injury are closely associated with the generation of ROS and/or reactive nitrogen species, which are major conditions leading to oxidative stress (Liu et al., 2004). The mitochondrial respiratory chain has long been described as the major source of ROS in cells. Peroxynitrite (PN) is generated by mitochondria and mediates oxidative stress in most but not all neurons (Bhat et al., 2015). After SCI, Ca^2+^ rapidly accumulates in the mitochondria, activating mitochondrial nitric oxide synthase to generate nitric oxide (NO·) (Filomeni et al., 2015). Simultaneously, electron leakage generates superoxide radicals (O_2_^–^·), which rapidly react to produce PN anions (ONOO^–^). ONOO^–^ can rapidly sequester H^+^ to produce PN. Simultaneously, the produced PN anions may interact with carbon dioxide to yield nitrosoperoxycarbonate (ONOOCO_2_^–^) and target the polyunsaturated fatty acids within the cell membrane, thereby compromising the membrane’s integrity and causing neuronal death (Heo and Lee, 2004). SCI has also been shown to decrease the activity of the physiological enzymatic antioxidant system, which includes superoxide dismutase and catalase as well as selenium-dependent glutathione peroxidase (Se-GPx, which occurs in selenium-dependent and selenium-free forms). Additionally, the activation of the immune system, along with the release of chemokines and cytokines, as well as an overexpression of numerous adhesion molecules on endothelial cells, can draw neutrophils and monocytes to the injury site, causing the release of substantial quantities of ROS and inflammatory mediators (Orr and Gensel, 2018). Conversely, due to the high concentrations of peroxidation-sensitive lipids, including arachidonic acid, linoleic acid, and docosahexaenoic acid, in the central nervous system, it is more vulnerable to oxidative stress (Magistretti and Allaman, 2015). As a result, the biological macromolecules of the central nervous system are more vulnerable to damage, such as protein deterioration and DNA breakage. In summary, oxidative stress causes neuronal damage through three main mechanisms: attacking the mitochondrial membrane close to where it is synthesized, which leads to mitochondrial dysfunction and increased production of ROS; interacting with lipids in the cell membrane, which causes lipid peroxidation and leads to structural changes and dysfunction of organelles and cell membranes; and interacting with nuclear DNA and proteins, which causes base modifications, DNA breaks, and protein fragmentation or degradation, which leads to DNA damage and protein dysfunction. (Catalá and Díaz, 2016; Yu et al., 2023). This process is illustrated in **Figure 1C**.

### Peripheral nerve damage

Although PNI shares some features with SCI, it also exhibits notable distinctions. Injured peripheral nerves undergo inflammatory reactions, oxidative stress, mitochondrial malfunction, and ion dysfunction, alongside the major degenerative process of peripheral nerve axons known as Wallerian degeneration, which occurs near the lesion site (Conforti et al., 2014). The axons at the lesion site deteriorate, while the distant axons experience a series of successive alterations. Initially, when peripheral nerves sustain injury, the cytoskeleton and cell membrane of the axons at the injury site fracture into minute pieces, and Schwann cells facilitate calcium influx, subsequently inducing protease secretion (Cashman and Höke, 2015). This event causes diminished impulse transmission and myelin degradation. Subsequently, the myelinated Schwann cells that have lost their innervation discard their myelin sheaths, proliferating in the basal layer and producing cytokines. These cytokines activate local macrophages and additionally recruit circulating macrophages to phagocytose the cellular debris resulting from the injury. These two cell types efficiently eliminate myelin and axonal debris while secreting substances to facilitate Schwann cell migration and axonal regeneration. After a period of time, the damaged axon develops a growth cone and initiates regeneration along the Büngner bands established by Schwann cells (Ribeiro-Resende et al., 2009; Panzer et al., 2020).

## Functions of Magnesium in the Repair of Brain Injury

In addition to the previously described functions of magnesium in the nervous system, magnesium ions also substantially contribute to brain activity. Magnesium ions in the brain facilitate nerve signal transmission and sustain ion homeostasis while also mitigating oxidative stress and preventing the release of vasoactive chemicals (Mathew and Panonnummal, 2021). Moreover, magnesium ions can safeguard the integrity and functionality of the blood–brain barrier, shielding the brain from toxins and pathogens (Romeo et al., 2019). Furthermore, magnesium ions are also strongly associated with inflammation in the brain. Because magnesium shortage causes neuroinflammation in the mouse brain by upregulating the expression of neuroinflammation-related genes in the hippocampus and cortex, studies have linked it to chronic low-grade inflammation in the brain (Maier et al., 2021; Tsuji et al., 2021). Magnesium also notably affects the brain through key inflammatory components of the central nervous system (NO and prostaglandins). Neuronal nitric oxide synthase is the primary producer of NO, a widely distributed gaseous cellular messenger (Zhou and Zhu, 2009). NO can modulate neuronal signaling and safeguard the CNS from ischemic injury (Faraci, 2006) and regulate neurotransmission and neuronal metabolism, among other functions (Picón-Pagès et al., 2019). Nonetheless, excessive production of NO is detrimental since NO can interact with superoxide to generate PN, which adversely affects lipids, proteins, and nucleic acids, while also enhancing the permeability of the blood–brain barrier (Lundberg and Weitzberg, 2022). This is due to the elevated activity of nitric oxide synthase under low magnesium circumstances (Maier, 2011; Kirkland et al., 2018). Prostaglandins, akin to NO, also perform local regulatory activities in the brain, including modulation of synaptic plasticity. Excessive production of prostaglandins in the brain not only induces and sustains inflammation but also impairs mitochondrial function, resulting in oxidative stress and cellular death (Lin and Beal, 2006). Furthermore, diminished magnesium ion levels may trigger nuclear factor (NF)-κB activation, establishing a strong correlation between magnesium insufficiency and inflammation.

Investigations of cerebral injuries have shown that brain injuries can be classified into different groups on the basis of their mechanism and etiology. The damage caused by neurodegenerative diseases such as Alzheimer’s disease and familial cerebral atrophy mostly results from the abnormal buildup of brain cells in these conditions. Injuries resulting from vascular illnesses encompass cerebral embolism, cerebral infarction, and similar conditions. Injuries resulting from trauma and infection include those caused by vehicular accidents as well as meningitis, brain abscesses, and cerebral empyema. This discussion mostly focuses on neurodegenerative disease-related injuries and traumatic brain injuries.

Neurodegenerative disorders, including Alzheimer’s disease, Parkinson disease, and multiple sclerosis, are among the most common ailments that impose a substantial cost on patients, their families, and society. These diseases share a common feature: disrupted magnesium levels (Maier et al., 2023). In patients with Alzheimer’s disease, the circulating magnesium content is much lower than that in healthy individuals (Du et al., 2022), and the microglia in the brain are highly active (Heneka et al., 2015), despite the disease’s complicated etiology. In an animal model of Alzheimer’s disease, inflammation and protein deposition reduced considerably after the addition of appropriate amounts of magnesium ions (Yu et al., 2015). These protective effects of magnesium can be explained by the fact that magnesium ions inhibit inflammation by downregulating IL-1β. The second-most common neurodegenerative disease, Parkinson disease, occurs as a result of a complex array of pathological events, including altered lysosomal function, endoplasmic stress, glutamine excitotoxicity, oxidative stress, abnormal protein aggregation, and neuroinflammation. Magnesium supplementation has shown promise in the treatment of Parkinson’s disease because research has shown that it can counteract the occurrence of excitotoxicity, reduce oxidative stress and neuroinflammation, and otherwise influence the course of Parkinson disease. Multiple sclerosis is a prevalent neurological disorder and a frequent cause of disability in young individuals. The characteristics of multiple sclerosis include demyelination and axonal degradation in various areas of the brain and spinal cord, along with disruption of the blood–brain barrier, which is predominantly driven by inflammatory activity (Filippi et al., 2018). Cytokines, nitric oxide, and mitochondrial dysfunction are major factors influencing illness initiation, recurrence, and progression. Magnesium supplementation is a viable therapeutic option for multiple sclerosis, considering the anti-inflammatory properties of magnesium ions and their contribution to mitochondrial function.

Furthermore, trauma-induced brain injury also requires attention. Traumatic brain injury can be classified into main and secondary phases. Initially, due to biomechanical injury, cerebral blood flow diminishes, resulting in hypoxia and hemorrhage that subsequently cause cerebral edema and elevated intracranial pressure (Stocchetti et al., 2007). The initiation of the primary phase is succeeded by an irreversible secondary phase, which is marked by disrupted calcium ion homeostasis, excessive release of glutamate, substantial production of ROS, and mitochondrial dysfunction. These changes collectively result in neurodegeneration (Fiskum, 2000; Lifshitz et al., 2003).

As stated previously, magnesium functions as a non-competitive inhibitor of NMDARs in standard physiological activities and is crucial for controlling calcium influx (Huang et al., 2025). Brain injury causes depletion of magnesium ions and the loss of homeostatic regulation over NMDARs, leading to a substantial influx of calcium. Furthermore, the decrease in magnesium ions facilitates the production of oxidative stress, as evidenced by a study involving patients with traumatic brain damage, wherein the administration of magnesium sulfate mitigated oxidative stress following such injuries (Cernak et al., 2000). Furthermore, a prospective clinical study conducted by Dhandapani et al. (2008) revealed that the death rate following brain injury was 13% among patients receiving magnesium treatment and 47% among those administered a placebo. Nonetheless, given the intricate factors influencing clinical findings, the applicability of magnesium supplementation in clinical practice requires further investigation. However, *in vitro* experiments have unequivocally demonstrated the role of magnesium ions in mitigating inflammation and oxidative stress following brain injury (Sen and Gulati, 2010). The protective effects of magnesium can be attributed to its ability to inhibit the expression of genes associated with neuronal apoptosis: specifically, TP53 mRNA upregulation was noted in the cortex, thalamus, and hippocampus following brain injury, whereas magnesium ions diminished TP53 expression (Lee et al., 2004).

In conclusion, while many obstacles remain in the clinical application of magnesium supplementation for the treatment of brain injury, its efficacy in enhancing recovery from such injuries is unequivocal. Consequently, magnesium ions can significantly contribute to the repair of cerebral injuries.

## Magnesium and Optic Nerve Injury

Optic nerve damage is defined by the degeneration of retinal ganglion cells and the elongation of axons (Kimura et al., 2017). Similar to SCI, optic nerve injury can also be categorized into primary and secondary injuries. Primary injury results from direct physical trauma, leading to axonal transection, cellular death, bleeding, ischemia, and other consequences. After the initial lesion, neuroinflammation and excitotoxicity contribute to secondary injury, ultimately resulting in the demise of optic nerve cells. During secondary damage, calcium ions, inflammatory cells, and released cytokines play crucial roles. The cytokines are involved in neuroprotection and substantially influence the outcome of damage (Liu et al., 2023). Consequently, comprehending the molecular pathways underlying optic nerve injury is essential for establishing axon-regeneration studies and prospective therapeutic modalities.

### Excitotoxicity

Analogous to SCI, damage to the optic nerve leads to diminished or disrupted blood flow to the affected region, resulting in heightened cell membrane permeability and the consequent influx and accumulation of calcium ions within the cells. The buildup of calcium ions subsequently triggers a significant release of excitatory neurotransmitters, including glutamate, which finally results in the demise of retinal ganglion cells. Furthermore, in contrast to SCI, damage to the optic nerve results in the release of a cytokine known as endothelin. Endothelin is a powerful vasoconstrictor that can induce retinal ischemia and is known to elevate intracellular Ca^2+^ levels in glial and neuronal cells. Moreover, endothelin can selectively induce glutamate efflux through the astrocyte’s endothelin receptors, aggravating the demise of retinal ganglion cells (Iezhitsa and Agarwal, 2018).

### Inflammatory reaction

The inflammatory response is a crucial component of the mechanism of optic nerve injury and is involved in all stages of such injuries. Microglia are stimulated by injury-associated signals, including alarmins and other damage-related molecular patterns, following optic nerve injury. In addition to removing debris, microglia also produce proinflammatory cytokines such as IL-1β, IL-6, and TNF-α, which attract immune cells to the injury site and enhance the immune response. Macrophages penetrate the wounded region 2–3 days post-injury, reaching their maximal concentration approximately 7 days after the injury. Macrophages also exhibit elevated nitric oxide synthase expression by activating the nuclear factor-κB signaling pathway, leading to increased neuronal death and the subsequent recruitment of additional inflammatory cells. The role of the inflammatory response in optic nerve injury is contentious, similar to that in SCI. Although inflammation mostly exhibits neurotoxicity, macrophages transition to an anti-inflammatory state in the advanced phases of optic nerve injury and secrete cytokines, which signal oligodendrocytes and neurons to facilitate axonal growth and myelin repair. Consequently, the functions of the inflammatory response in optic nerve injury cannot be easily delineated (Liu et al., 2023).

### Magnesium in the management of optic nerve injury

As mentioned above, glutamatergic excitotoxicity is mainly caused by the intracellular accumulation of calcium ions. Due to their relatively high permeability to calcium ions, N-methyl-D-aspartate (NMDA)-type glutamate-gated channels render neurons especially vulnerable to damage from excessive activity of this channel subtype. The NMDAR was initially identified in the retina by Lucas and Newhouse (1957) (Lambuk et al., 2017), and retinal ganglion cells are recognized for expressing NMDA-type channels. Consequently, from a therapeutic perspective, the NMDAR represents a viable target for interventions to avert retinal ganglion cell degeneration. In this regard, magnesium ions represent an excellent therapeutic option. Lambuk et al. (2017) used magnesium acetyltaurate to provide neuroprotection against the excitotoxicity induced by NMDARs in rat retinas. Their findings indicated that intravitreal magnesium acetyltaurate pretreatment inhibited the death of retinal ganglion cells triggered by NMDARs. This study suggests that both magnesium and taurine may be involved in the protective effects on retinal ganglion cells, although the specific contributions of each component were not examined. Arfuzir et al. (2016) used magnesium acetyltaurate to safeguard the retina and optic nerve against endothelin-induced injury. Their findings indicated that magnesium acetyltaurate pretreatment in the vitreous body diminished caspase-3 activation and averted endothelin-induced retinal cell injury and axonal degeneration. Conversely, other studies (López-Riquelme et al., 2015; Lambuk et al. 2019) have shown that elevated endothelin-1 levels are correlated with subclinical inflammation, and that ET-1-induced ischemia and hypoxia enhance the generation of reactive oxygen and nitrogen species, resulting in prolonged oxidative and nitrosative stress, which is linked to neuroinflammation. In the study by Arfuzir et al. (2016), magnesium acetyltaurate was employed to mitigate neuroinflammation in SD rats, thereby averting retinal ganglion cell loss induced by endothelin-1. The findings indicated that magnesium acetyltaurate pretreatment may inhibit the expression of inflammatory mediators, including IL-1β, IL-6, TNF-α, and NF-κB, in the retinas of ET-1 induced rats, and enhance the survival rate of retinal ganglion cells.

In conclusion, while acetyl sulfoxide exhibits neuroprotective properties, the protective role of magnesium ions on the visual nerve is substantial and cannot be overlooked. Magnesium ions are crucial for the healing of optic nerve injury.

## Applications of Magnesium in the Repair of Spinal Cord Injury

As mentioned above, magnesium ions are endogenous antagonists of voltage- and ligand- gated calcium channel glutamate-NMDARs, which can reduce the excitotoxicity caused by calcium overload after nervous system injury and reduce neuronal cell death. Simultaneously, magnesium serves as a crucial cofactor in the energy-production mechanisms of the tricarboxylic acid cycle and oxidative phosphorylation, which can sustain mitochondrial function and rectify the energy imbalance following SCI. Moreover, magnesium can modulate intracellular calcium concentrations in vascular endothelial cells by blocking calcium channels. It diminishes endothelin-1 synthesis and obstructs myosin light chain kinase activation, thereby mitigating vascular spasm and contraction while enhancing cerebral blood flow (Kaptanoglu et al., 2003a; Kirkland et al., 2018).

Prior research indicates that during the secondary injury phase following traumatic SCI, oxidative stress and neuroinflammation at the injury site are critical determinants of injury severity and may serve as possible therapeutic targets. Wiseman et al. (2009) investigated and assessed the therapeutic efficacy of magnesium sulfate following spinal cord damage. Their findings indicated that, in comparison to the control group, the Basso-Betty-Bressnahan (BBB) score of the magnesium-treated group was significantly superior; additionally, the white matter and total cross-sectional area preserved in the lesion center in the magnesium-treated group were more substantial relative to the corresponding findings in the control group. However, the number of model mice used in their experiment was relatively small, and the possibility of errors in comparison with steroid treatment could not be ruled out. In addition, Kaptanoglu et al. (2003b) also studied the effects of magnesium sulfate on ultrastructural changes after SCI and evaluated early clinical treatment effects. Their findings showed notable axonal structural damage in the trauma group, with the majority of myelin sheath layers torn, the axoplasm of the axons exhibiting a scarcity of mitochondria, and substantial edema gaps prevailing within the cytoplasmic structure. In comparison with the trauma group, the neuron nuclei in the group administered 600 mg/kg magnesium sulfate exhibited denser chromatin and intact nuclear membranes, with the majority of axons showing preservation of normal neurofilament content, the mitochondria remaining largely unharmed, and the myelin layers remaining predominantly intact. Moreover, in terms of early clinical treatment outcomes, the Tarlov and BBB scores of the 600-mg/kg magnesium treatment group were most comparable to those of the control group and markedly superior to those of the trauma group. Although the study by Kaptanoglu et al. (2003b) verified the neuroprotective effect of high-dose magnesium in acute SCI, it did not determine the most effective dosing regimen in humans, so further pharmacokinetic and dose-response studies should be conducted.

In conclusion, magnesium treatment was highly effective in preserving spinal cord ultrastructure. However, additional studies are required to further evaluate the potential benefits of magnesium therapy in managing acute SCI in humans.

The aforementioned experiments were performed by researchers utilizing mice, and their findings are not directly applicable to humans. Sperl et al. (2019) performed a prospective clinical observational study to examine the relationship between fluctuations in serum magnesium content and the remission of neurological function in individuals with traumatic spinal cord damage. Their findings indicated that reduced magnesium levels in the initial phase following spinal cord damage may correlate with enhanced neurological recovery, whereas elevated magnesium levels are linked to inferior outcomes. Patients exhibiting a rapid decline in magnesium levels post-injury were more likely to regain neurological function, whereas persistently elevated magnesium levels were correlated with heightened inflammation and diminished recovery. This finding indicates that a specific concentration of magnesium ions is beneficial for SCI healing. However, this study is not without limitations, of which the primary limitation was the relatively small size of the patient group. Therefore, the results should be interpreted as suggestive, and a larger population is needed to draw more precise conclusions.

Xie et al. (2022) used poly (l-lactide-co-ε-caprolactone) (PLCL) to coat magnesium oxide nanoparticles to treat SCI. Their findings indicated that magnesium ions can inhibit calcium inflow. Furthermore, the neuroprotective influence of magnesium ions *in vivo* was confirmed, revealing a significant reduction in apoptotic cells in the group administered magnesium oxide nanoparticles, alongside the largest count of neural stem cells identified with nestin.

In conclusion, while utilization of biological tissue engineering involving magnesium ions for SCI healing remains constrained, the substantial potential of magnesium ions in treating spinal cord injuries merits additional investigation.

## Application of Magnesium in the Repair of Peripheral Nerve Injury

As stated previously, biological tissue engineering technology has attracted increasing attention in the treatment of PNI due to its distinctive advantages. A growing number of magnesium-based materials have been identified to be useful for peripheral nerve repair owing to their distinctive capacity to enhance nerve regeneration, making them important candidates for nerve conduits and scaffolds. The released Mg^2+^ can enhance neurite development in a concentration-dependent manner by activating the phosphoinositide 3-kinase (PI3K)/protein kinase B (Akt) signaling pathway and Sema5b (Yao et al., 2022). Furthermore, magnesium ions possess anti-inflammatory effects and stimulate Schwann cells to release nerve growth factor (Bachnas et al., 2022). Consequently, regardless of whether they are produced by magnesium metal, magnesium alloy, magnesium-based metallic glass (MG), or surface-modified magnesium materials, the magnesium ions produced during their degradation process are crucial in facilitating nerve regeneration. This section provides a comprehensive overview of the magnesium-based materials utilized in the treatment of PNIs (**Additional Table 1**).

**Additional Table 1:** Various magnesium-based materials used for repair of peripheral nerve injury

Material type	Feature	Application advantage	Potential challenge	Reference
Magnesium metal	Excellent biocompatibility and degradability.	It can provide mechanical support and its degradation will release soluble Mg^2+^ and hydrogen, both of which have neuroprotective and antioxidant properties. High conductivity provides short-term electrical stimulation for nerve growth.	The degradation rate is too fast and cannot support the complete repair of nerve damage.	Hopkins et al. (2017) used magnesium wire as the inner core of a nerve conduit to repair short-gap and long-gap peripheral nerve injury. Zhang et al. (2021b) woven metal magnesium into the inner layer of nerve guide catheters for repair of injuryed peripheral nerve. Li et al. (2016a) used magnesium wire to repair damaged sciatic nerve.
Magnesium alloy	More sturdy. Stronger electrical conductivity. The degradation rate is more controllable. Other metal elements (such as Li) can form alloys with aagnesium.	It can provide good structural support. It can enhance the electrical stimulation or signal conduction of nerve cells. Other metal elements that are beneficial to nerve regeneration can work together with magnesium to promote nerve repair.	For the repair of long-gap nerve damage, its degradation rate still cannot meet the needs of nerve growth. Poor plasticity. The hydrogen charging and discharging window is narrow. It is rarely used in the field of nerve repair.	Fei et al. (2017) evaluated the biocompatibility and neurotoxicity of three alloys: Mg-2Nd-Zn, Mg-2Zn-Nd and rolled Mg-10Li. Bhat et al. (2024) investigated the biocompatibility and neuroinflammatory response of glial cells through *in vitro* studies utilizing magnesium-lithium films.
Surface modified magnesium- based materials	Can be covered with a variety of coatings.	The new coating can slow down the degradation rate of magnesium metal to better adapt to nerve regeneration. The new layer can provide more functions, such as anti-inflammatory and anti-proliferative funtions.	The modified materials may not be suitable for cell adhesion and growth and cannot meet the flexibility required for nerve growth. It is rarely used in the field of nerve repair.	Almansoori et al. (2018) used a magnesium alloy covered with a layer of hydroxyapatite to prepare a nerve guide catheter to promote the repair of damaged nerves. Liu et al. (2012) prepared a magnesium alloy coated with carbon nanotubes/calcium phosphate/chitosan to promote the growth of rat dorsal root ganglion neurons.
Magnesium- based metallic glass	Higher strength and lower Young's modulus. Better wear resistance, corrosion resistance and good fatigue performance.	Can provide long-term structural support for nerve repair.	From a processing perspective, it is still very difficult to obtain samples large enough for use. It is rarely used in the field of nerve repair.	Li et al. (2016c) added Sr to the Mg-Zn-Ca system and observed that MC3T3-E1 preosteoblasts adhered well and grew healthily on the surface of Mg-Zn-Ca metallic glass. Monfared et al. (2018) prepared ribbon-shaped Mg70Zn26Ca4 metallic glass by melt spinning and selected Schwann cells s as the initial material for evaluating biocompatibility in neural tissue applications.

### Magnesium

Magnesium metal has been extensively researched in the biomedical domain in the last few years due to its superior biocompatibility and degradability. Magnesium, as a metal, offers mechanical support and releases soluble Mg^2+^ and hydrogen gas upon degradation, both of which possess neuroprotective and antioxidant effects (Takeuchi et al., 2015). Moreover, the elevated electrical conductivity of magnesium metal facilitates short-term electrical stimulation for neuron regeneration, which is advantageous for nerve growth. Hopkins et al. (2017) used magnesium wire as the inner core of nerve conduits for healing both short and long gaps in peripheral nerves in a study. The findings indicated that during 6 weeks of short-gap repair, the inclusion of Mg metal wire facilitated axonal nerve regeneration and increased the total number of axons. Although additional examination of myelin characteristics is required to validate the augmentation in axonal volume, the enlargement of axonal size is unequivocal. Despite the absence of functional enhancement or enhanced axonal development in long-gap repair, the incorporation of Mg wire significantly ameliorated tissue inflammation. In addition, Zhang et al. (2021b) wove magnesium into the inner layer of nerve guide catheters for peripheral nerve repair, and used the sciatic function index (SFI) to evaluate the treatment effect. The findings indicated that the SFI of the catheter group was markedly distinct from that of the defect group (*P* < 0.01), but it closely aligned with that of the autologous transplantation group. Vennemeyer et al. (2015) used magnesium to rehabilitate the injured sciatic nerve in rats. The experimental findings indicated that all magnesium wires were centrally positioned and entirely encircled by regenerated tissue, suggesting that magnesium could function as a scaffold for cellular proliferation. The tissue tolerance to magnesium metal was evident, with the findings showing modest inflammation, significant nerve regeneration in histological analysis, and the absence of necrosis. Similar to the approach employed by Vennemeyer et al. (2015), Li et al. (2016a) used magnesium wire to mend the injured sciatic nerve and subsequently examined the cross-section of the distal nerve stump after a designated period using NF200 and S100 staining. Enhanced axon regeneration and myelin sheath (NF200- and S100-positive) coverage were noted in the injury + magnesium group in comparison with the damage group (Li et al., 2016a). These findings imply that magnesium metal can facilitate nerve regeneration.

### Magnesium alloy

The healing of peripheral nerves is a protracted procedure that necessitates directional supervision. Consequently, while magnesium metal has been utilized in PNI treatment, it shows excessively rapid degradation, resulting in complete disintegration prior to the completion of nerve healing, which presents a major challenge. In this regard, research attention has increasingly focused on the use of magnesium alloys. In comparison to conventional magnesium metal, magnesium alloys offer the following advantages: They possess more strength than pure magnesium metal and can offer substantial structural support. Magnesium alloys also exhibit superior conductivity and can improve nerve cells’ electrical stimulation or signal transmission. The deterioration rate of magnesium alloys is also more manageable. Other metallic elements advantageous for nerve regeneration, such as lithium, can alloy with magnesium to facilitate nerve restoration. The utilization of magnesium alloys in orthopedic devices and cardiovascular stents has demonstrated its practicality in application. Consequently, magnesium alloys are expected to serve as efficient materials for nerve healing.

In biomedical applications, common alloys include Mg-Ca-based, Mg-Zn-based, and Mg-Li-based alloys. Calcium is the main component of human bones and has good biocompatibility. It is also one of the most studied additives for the development of biodegradable Mg alloys (Azadi et al., 2025). Zinc is an essential nutrient for the human body that promotes growth and enzyme synthesis and stimulates healing. It is a common alloying component in magnesium alloys for solid solution strengthening (Loukil, 2021). Lithium is the main drug for treating psychiatric diseases. It also has anti-inflammatory and neuroprotective effects and offers broad development prospects in the synthesis of magnesium-based alloys.

Among studies using magnesium alloys in the repair of peripheral nerve damage, Fei et al. (2017) evaluated the biocompatibility and neurotoxicity of three alloys: Mg-2Nd-Zn (NZ20), Mg-2Zn-Nd (ZN20), and rolled Mg-10Li. Extracts from these three alloys were utilized to replicate the microenvironment following magnesium alloy implantation. The findings indicated that the extracts of the three alloys exhibited minimal cytotoxicity in the short term (1 day). However, the NZ20 alloy extract negatively influenced cell proliferation over a more extended duration (3 days). The pattern on the fifth day was approximately identical to that on the third day. Moreover, for neurotoxicity, the Mg-10Li extract exhibited the lowest neurotoxicity, followed by the ZN20 extract. The NZ20 extract demonstrated significant neurotoxicity, presumably due to the elevated ion concentration during breakdown. Although not conducted *in vivo*, this study identified the Mg-10Li alloy as a potentially viable biodegradable magnesium alloy for the healing of PNI. The breakdown rate of magnesium alloys is crucial for facilitating nerve regeneration. Similar to Fei et al. (2017), Bhat et al. (2024) studied the biocompatibility and neuroinflammatory response of glial cells *in vitro* using magnesium-lithium films. Their results showed that both cell types proliferated well regardless of the Li concentration, and in the mouse brain slice model, Mg-1.6 wt%Li and Mg-9.5 wt%Li alloy extracts did not cause significant upregulation of glial inflammatory/reactive markers (IL-1β, IL-6, fibronectin 1, tenascin C) after 24 hours of exposure. These results indicated that the two Mg-Li thin film alloys have excellent cell compatibility and provide a basis for future research on neural repair applications. However, Bhat et al. (2024) conducted *in vitro* experiments. The *in vivo* environment is more complex and dynamic than the *in vitro* environment, which may lead to corrosion rates that are completely different and different results. Therefore, further exploration is needed.

Although few studies have focused on the use of pure magnesium alloys to repair PNIs (Fei et al., 2017; Bhat et al., 2024), Fei et al. (2017) reported that the design ratio of magnesium alloys is closely related to their degradation rate and biological toxicity. The study by Bhat et al. (2024) shows that the *in vivo* and *in vitro* environments will also affect their degradation rate and biological toxicity. These findings have undoubtedly laid a solid foundation for the use of magnesium alloys in repairing PNIs.

### Surface-modified magnesium materials

Despite advancements in the degradation rate of magnesium alloys, the degradation rates still remain inadequate to support nerve growth for the repair of extensive nerve damage. Moreover, magnesium alloys’ comparatively low strength, inadequate flexibility, and restricted hydrogen absorption and desorption range constrain their biomedical applications (Krüger et al., 2022; Yang et al., 2023). To address these issues, surface modification has increasingly gained attention (Guan et al., 2022). For instance, Sun et al. (2021) applied a poly (L-lactide)/paclitaxel coating to the surface of Mg-Zn-Y-Nd alloy stents to enhance their corrosion resistance in physiological conditions; conversely, Sun et al. (2019) utilized a fluoride coating on the surface of magnesium alloy to mitigate its degradation rate. Furthermore, Li et al. (2020) fabricated a PDA/HA coating on the surface of the Mg-Zn-Y-Nd alloy. The experimental findings indicated that this coating markedly enhanced the corrosion resistance of magnesium alloy. Furthermore, the PDA/HA coating augmented the anti-proliferative and anti-inflammatory properties of magnesium alloy.

In one of the studies using modified magnesium materials to treat damaged peripheral nerves, Almansoori et al. (2018) used magnesium alloys coated with a layer of hydroxyapatite to prepare nerve guide catheters to promote the repair of damaged nerves. Three months post-implantation, the coated magnesium alloy exhibited minimal absorption, with no gas production observed in the adjacent tissues. The proliferation of PC12 cells in the coated magnesium alloy group was much greater than in the control group. The coated magnesium alloy group also showed fewer axons in terms of tissue morphology. The authors concluded that the catheter surface was excessively coarse or that the catheter lacked sufficient flexibility. Similar to Almansoori et al. (2018), Liu et al. (2021) prepared a magnesium alloy coated with carbon nanotubes/calcium phosphate/chitosan to promote the growth of rat dorsal root ganglion neurons. The quantity of axons and branches was monitored over a specified duration. The findings indicated that the magnesium alloy group coated with carbon nanotubes, calcium phosphate, and chitosan exhibited the highest quantity of axons and branches after a designated period. The researchers also examined the impact of the extract from the coated magnesium alloy on the release of nerve growth factors and discovered that the concentrations of nerve growth factor and brain-derived neurotrophic factor in the cell culture supernatant of the coated magnesium alloy group were markedly elevated in comparison to the control group.

While the applications of surface-modified magnesium-based materials in peripheral nerve healing are limited, surface modification alone is insufficient for effective nerve repair. Other important considerations include the changed material’s suitability for cell adhesion and proliferation and its compliance with the flexibility necessary for nerve growth. Simultaneously, studies have shown that applying a functionally specialized coating to the material’s surface can enhance the regeneration of peripheral nerves. Consequently, surface-modified magnesium-based materials offer significant potential for healing PNIs (Liu et al., 2021; Tatu et al., 2023).

### Magnesium-based metallic glass

In contrast to conventional crystalline metals, MGs, also referred to as amorphous or glassy alloys or liquid metals, represent a novel category within the metallurgical domain. The amorphous structure of MG imparts distinctive features to these materials, including enhanced strength, reduced Young’s modulus, superior wear and corrosion resistance, and commendable fatigue performance (Meagher et al., 2016). Ti-, Zr-, and Fe-based MGs have been examined as non-biodegradable materials for biological purposes. Conversely, Mg-, Ca-, Zn-, and Sr-based MGs represent a category of biodegradable amorphous alloys that have been examined to far (Li et al., 2016b). In fact, Mg-based MGs have gained attention as a new class of biodegradable magnesium alloys with suitable properties for biomedical applications because of their excellent corrosion resistance. In this regard, Mg-based MGs, including ribbon or bulk Mg-Zn-Ca, Mg-Cu-Gd, Mg-Ni-Nd, and Mg-Cu-Y systems, have been successfully developed, with the Mg-Zn-Ca metallic glass system garnering particular attention due to its superior glass-forming ability and the presence of vital elements Ca and Zn *in vivo* (Dambatta et al., 2015). Furthermore, Gu et al. (2010) demonstrated that MG63 cells show strong adhesion and proliferation capabilities on the Mg66Zn30Ca4 surface.

The quick breakdown of magnesium complicates the repair of extensive nerve gaps in PNIs, hindering complete regeneration. Due to its corrosion resistance, Mg-based MG may serve as an effective alternative to crystalline alloys in nerve tissue applications for healing of long nerve gaps. Li et al. (2016d) conducted a study in which Sr was incorporated into the Mg-Zn-Ca system, and their findings showed favorable adhesion and healthy proliferation of MC3T3-E1 preosteoblasts on the surface of the Mg-Zn-Ca MG. Moreover, Schwann cells play a crucial role in peripheral nerve regeneration by secreting neurotrophic chemicals, producing cell adhesion molecules, and creating a conducive environment for regenerating axons. In the study conducted by Monfared et al. (2018), ribbon-shaped Mg70Zn26Ca4 MG was synthesized using melt spinning, and Schwann cells were chosen as the preliminary material for biocompatibility assessment in nerve tissue applications. The findings indicated that the Mg70Zn26Ca4 glass ribbon facilitated Schwann cell adhesion, and the Mg70Zn26Ca4 extract exhibited minimal cytotoxicity toward Schwann cells, demonstrating superior biocompatibility in comparison with pure magnesium.

Despite the superior anti-degradation capabilities, enhanced mechanical characteristics, and biocompatibility of Mg-based MG, research on the biomedical applications of Mg-based bulk MG remains nascent. Monfared et al. (2018) only assessed the biocompatibility of Mg70Zn26Ca4 ribbon MG utilizing Schwann cells, and no research group has investigated the direct application of MG for the repair of peripheral nerve damage. Moreover, from a processing standpoint, acquiring sufficiently large samples remains exceedingly challenging. Nevertheless, the research by Monfared et al. (2018) indicates that Mg-based MG inside the Mg-Zn-Ca system is a promising biomaterial that holds substantial promise for nerve restoration in tissue engineering.

For repair of peripheral nerve damage, this article provides a concise overview of the properties and benefits and drawbacks of several magnesium-based materials, including MG, surface-modified magnesium-based materials, alloys, and metal magnesium. These findings will potentially shed fresh light on future uses for a variety of magnesium-based compounds in the treatment of PNIs.

## Role of Magnesium Ions

As stated previously, magnesium ions serve a crucial regulatory function in neurotransmitter release and synaptic transmission under typical physiological settings. Damage to the nervous system disrupts local magnesium ion homeostasis, impairing the ions’ regulatory role in transmitter release. This results in excitotoxicity and mitochondrial damage, causing secondary injury at the site of nerve damage. Consequently, preserving magnesium homeostasis at the injury site is crucial for regeneration of damaged nerves. Moreover, magnesium ions show neurorepair and neuroprotective properties. Magnesium ions can contribute to neurorepair by facilitating the synthesis of membrane phospholipids, myelin, and synapses, while also promoting axonal development and the proliferation of neural stem cells (Kim et al., 2025b). Magnesium ions provide neuroprotection by preventing excitotoxicity in nerve cells, safeguarding mitochondrial activity, diminishing oxidative stress, and combating neuroinflammatory responses. This section provides a comprehensive introduction to the neuroprotective role of magnesium ions (**Additional Table 2**).

**Additional Table 2:** Effects of magnesium ions

Role of magnesium ions	Specific function	Mechanism	Reference
Against excitotoxicity	Reduce excessive release of excitatory neurotransmitters (such as glutamate).	Magnesium ions regulate NMDA receptor activity, thereby reducing neuronal damage caused by intracellular calcium overload.	Gougoulias et al. (2004) found that systemic treatment with magnesium can prevent NMDA receptor-mediated excitotoxicity caused by nerve damage. Lambuk et al. (2017) found that magnesium acetyltaurine has a protective effect on NMDA receptor-mediated excitotoxicity in rat retina.
Anti-inflammatory effects	Relieve excessive inflammatory response after nerve damage.	Inhibit the NF-kB signaling pathway and reduce the production of pro-inflammatory factors (such as TNF-α and IL-1).	Xie et al. (2019) found that magnesium ions can inhibit the NF-kB signaling pathway and reduce the expression of pro-inflammatory factors, thereby reducing inflammatory responses. Veronese et al. (2022) proved that magnesium ions can reduce the release of inflammatory factors, but it is selective.
Reduce oxidative stress and protect mitochondria	Antioxidant effect, reducing free radical damage Maintaining normal energy metabolism of mitochondria.	Magnesium ions can regulate the activity of antioxidant enzymes in the body. Magnesium ions can reduce ROS generated by oxidative stress reactions. Magnesium ions can regulate the activity of calcium uniporters on the mitochondrial membrane and reduce the excessive accumulation of calcium ions in mitochondria.	Lopez-Baltanas et al. (2023b) and Mohammadi et al. (2020) used the experimental model of metabolic syndrome of renal failure and the experimental model of hypoxia to verify the role of magnesium ions in reducing oxidative stress. Fujita et al. (2023) and Vida et al. (2021) found that magnesium ions can reduce oxidative stress in cells. Ponnusamy et al. (2024) found that magnesium ions are a resistor of mitochondrial calcium uniporter, which can regulate and prevent excessive calcium ions from entering mitochondria.

This table shows the neuroprotective benefits of magnesium ions in promoting nerve injury recovery and repair. These benefits include anti-inflammatory effects, anti-excitotoxic properties, mitochondrial protection, and the reduction of oxidative stress. This table offers new insights into how magnesium ions help prevent agaist nerve injury by outlining their specific roles and mechanisms. IL-1: Interleukin-1; NF-kB : nuclear factor kappa B; NMDA: N-methyl-D-aspartate; ROS: reactive oxygen species; TNF-α: tumor necrosis factor alpha.

### Fighting excitotoxicity

Glutamate is the predominant neurotransmitter in the mammalian central nervous system and facilitates excitatory communication between neurons. Glutamate frequently attaches to calcium-permeable ionotropic receptors, primarily NMDARs. Upon glutamate binding to the receptor, the ionotropic glutamate receptor facilitates the influx of calcium and sodium ions through its ion channel activity, resulting in the associated action. However, excessive glutamatergic input can induce excitotoxicity, resulting in neuronal cell death. Abnormal amounts of glutamate or other excitatory amino acids accumulate outside the cell, resulting in overstimulation of glutamatergic receptors and an abnormal influx of calcium ions into the cell.

Magnesium ions are antagonists of calcium ions and play a vital role in synaptic plasticity. Numerous studies have indicated that, under typical conditions, synaptic activity results in the release of Mg^2+^ from this channel, facilitating the entry of Ca^2+^ into neurons and the activation of subsequent intracellular signaling pathways (Bliss and Collingridge, 1993; Fekete and Wang, 2025; Huang et al., 2025). Upon the completion of signal transmission, Mg^2+^ obstructs the NMDAR channel, inhibiting excessive influx of Ca^2+^ into neurons.

As mentioned earlier, upon injury to the neurological system as a result of ischemia and hypoxia, the elevated cell membrane permeability at the wound site results in a substantial buildup of calcium ions, which eventually causes irreversible harm. Magnesium ions may mitigate the harm induced by ischemia and hypoxia. Gougoulias et al. (2004) observed that systemic application of Mg could attenuate NMDAR-mediated excitotoxicity. Lambuk et al. (2017) determined the neuroprotective effects of intravitreal magnesium acetyltaurinate against NMDA induced retinal excitotoxicity in rats, and found that administration to rats markedly prevented retinal cell degeneration under direct exposure to NMDA. Moreover, pretreatment showed better protective effects than co- or post-treatment. Conversely, Pradhan et al. (2011) developed a mathematical model based on the known kinetic properties of unidirectional transporters and the inferred mechanisms of inorganic phosphate regulation and magnesium ion inhibition. Their findings showed that the calcium ion absorption kinetics were S-shaped when magnesium ions were present and hyperbolic when they were not, indicating that magnesium ions inhibited calcium ion absorption.

To summarize, magnesium ions play a crucial role in controlling mineral homeostasis within cells (via preventing calcium influx), and this property substantially mitigates excitotoxicity.

### Anti-inflammatory effects

The inflammatory response is closely related to the Mg^2+^ ion. Lack of magnesium ions initiates an inflammatory response through the following pathways: (1) neutralizing the blockage of NMDARs induced by magnesium ions, leading to hyperactivation and activation of substance P; (2) activating NF-κB-dependent pathways, leading to high expression levels of inflammatory molecules; and (3) inhibition of the NF-κΒ signaling cascade causes degeneration of the expression for anti-inflammatory compounds nitric oxide (NO), protectins, lipoxins, and resovlins (Liu and Dudley, 2020).

Early inflammation can help clear damaged debris after nervous system injury and allow the regrowth of injured nerve. Prolonged inflammatory responses can inflict additional harm on nerve tissue, primarily due to the hyperactivation of glial cells and the excessive secretion of inflammatory mediators such as TNF-α, IL-6, and IL-1.

Regarding the role of magnesium ions in reducing neuroinflammation, some studies (Sugimoto et al., 2012; Maier et al., 2021; Khatib et al., 2022) have shown that magnesium ions can inhibit the NF-κB signaling pathway and thus reduce the expression of proinflammatory factors, thereby lowering the overall inflammatory response in the brain. These findings represent compelling data on the role of magnesium as an NF-κB inhibitor. Furthermore, the study conducted by Xie et al. (2019) corroborated this assertion. Veronese et al. (2022) conducted a meta-analysis and systematic review of clinical studies on the anti-inflammatory properties of magnesium ions. The results showed that whereas IL-8, IL-10, IL-17, erythrocyte sedimentation rate, and serum amyloid levels did not change significantly, magnesium ions significantly reduced plasma fibrinogen, tartrate-resistant acid phosphatase type 5, tumor necrosis factor ligand superfamily member 13B, tumorigenicity 2 protein, and IL-1. Magnesium ions are thought to specifically lower inflammatory factors. Magnesium also plays a major role in neurological conditions. According to an Alzheimer’s disease study, magnesium supplements can decrease levels of pro-inflammatory factors and increase levels of anti-inflammatory mediators in the hippocampal regions of rat models of Alzheimer’s disease. Furthermore, magnesium ion supplementation may diminish the prevalence of infant cerebral palsy (Sugimoto et al., 2012). In conclusion, magnesium ions exhibit anti-inflammatory effects in neurological disorders and *in vitro* animal models, potentially through the suppression of the NF-κB signaling pathway.

### Mitochondrial protection and reduction of oxidative stress

The production of ROS is closely linked to mitochondria, which serve as the body’s factories for ATP synthesis. Under typical physiological circumstances, ROS function as signaling messengers in intracellular signal transduction and are involved in both innate and adaptive immune responses, gene expression regulation, and programmed cell death or necrosis (Schieber and Chandel, 2014). However, damage to nerves causes an imbalance between the antioxidant and oxidative systems of the cell, with the oxidative system taking over. The resultant excess production of ROS interferes with redox signal transduction and damages molecules. Mitochondria are the main source of ROS in cells during this process. Increased mitochondrial Ca^2+^ levels cause the membrane to become more permeable to Ca^2+^, which activates several lipolytic and proteolytic enzymes. These changes reduce ATP levels and damage the membrane. When a critical threshold is reached, Ca^2+^ causes the mPTP to open continuously, which reduces the inner membrane potential of the mitochondria and stops the synthesis of ATP. The cells and mitochondria consequently disintegrate.

The influx of calcium ions into mitochondria necessitates calcium uniporters, and magnesium ions can modulate the function of these uniporters, thereby influencing the concentration of calcium ions within mitochondria. Ponnusamy et al. (2024) observed that magnesium ions act as inhibitors of mitochondrial calcium uniporters, preventing the influx of excessive calcium ions into mitochondria.

Similar to inflammatory responses, magnesium deficiency can also lead to oxidative stress. Intracellular magnesium deficiency inhibits magnesium ion transport to the mitochondria via mitochondrial RNA splicing protein 2 (MRS2) and facilitates magnesium ion efflux from mitochondria through solute carrier family 41 member 3 (SLC41A3), causing a reduction in mitochondrial magnesium ion concentration.

A deficit of magnesium in the mitochondria diminishes the functionality of the ETC, altering coupled respiration and elevating the generation of mitochondrial ROS (Gout et al., 2014). Moreover, the activation of mitochondrial channels resulting from magnesium shortage (Bednarczyk et al., 2005) and the stimulation of the renin-angiotensin-aldosterone system (Ahokas et al., 2005) are associated with the generation of ROS.

A growing body of studies has evaluated the use of magnesium ions in mitigating oxidative stress. Rajkumar et al. (2022) employed gelatin/polyvinyl alcohol-infused magnesium hydroxide nanocomposites to address neurotoxicity and oxidative stress in rats with Alzheimer’s disease. In their study, gelatin/polyvinyl alcohol was utilized to administer magnesium hydroxide nanoparticles to treat neurotoxicity and oxidative stress, resulting in a considerable reduction of mitochondrial ROS intensity in SH-SY5Y cells. Although the findings did not explicitly characterize the involvement of magnesium ions, this experiment demonstrated the general importance of the magnesium hydroxide molecule and indicates the importance of magnesium ions in mitigating oxidative stress. López-Baltanás et al. (2023) and Mohammadi et al. (2020) used experimental models of renal failure, metabolic syndrome, and hypoxia to investigate the effect of magnesium ions in mitigating oxidative stress. However, López-Baltanás et al. (2023) utilized oral supplementation, whereas Mohammadi et al. (2020) used intraperitoneal injection. López-Baltanás et al. (2023) observed that magnesium supplementation can enhance the activity of glutathione peroxidase and lipid peroxide in the body; however, this effect was contingent on the concentration of magnesium ions, and the levels of the two antioxidants exhibited a negative correlation. Likewise, Mohammadi et al. (2020) observed that irrespective of the hypoxia mode, the administration of magnesium ions at concentrations of 500 and 600 mg/kg resulted in a reduced level of the lipid peroxidation marker malondialdehyde in the mitochondria in comparison with the control group. This finding indicated that magnesium ions exert a concentration-dependent influence in mitigating oxidative stress in the organism. Furthermore, Fujita et al. (2023) and Vida et al. (2021) observed that magnesium ions can diminish intracellular oxidative stress.

**Figure 2:**
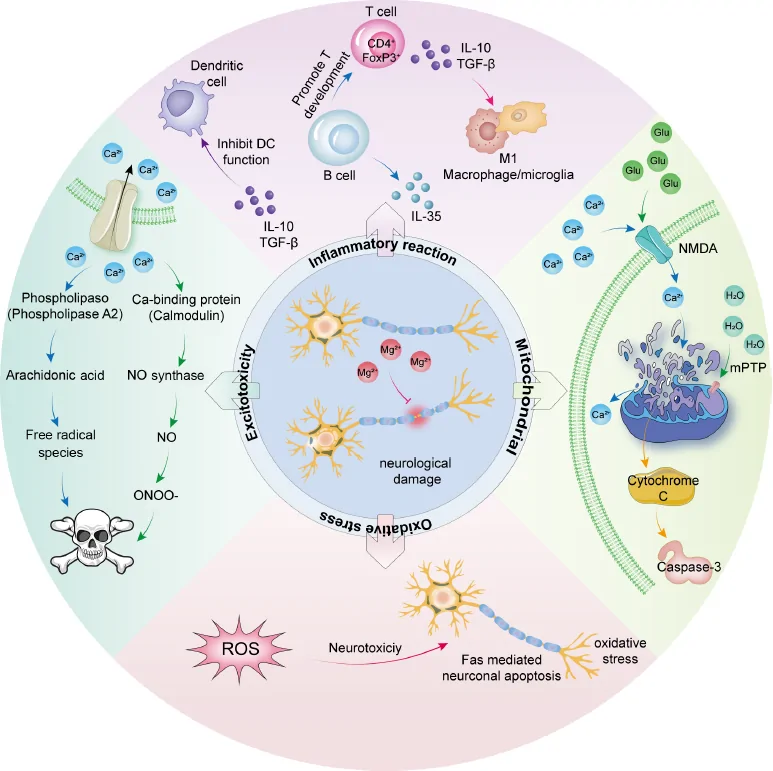
Summary of nerve injury mechanisms and the role of magnesium ions. Following nerve damage, oxidative stress, excitotoxicity, inflammatory reactions, and mitochondrial damage may occur. Magnesium ions play a crucial role in nerve injury repair. DAMP: Damage-associated molecular patterns; mPTP: mitochondrial permeability transition pore; NMDA: N-methyl-D-aspartate; NO: nitric oxide; O_2_^–^: superoxide radicals; ONOO^–^: peroxynitrite anions; ROS: reactive oxygen species.

In summary, excitotoxicity, inflammatory response, oxidative stress, and mitochondrial dysfunction all contribute to the damage process, regardless of whether the injury is to the central nervous system (spinal cord, brain), optic nerves, or peripheral nerves. However, as an essential trace element in the human body, magnesium ions have particularly strong neuroprotective effects. Through a variety of mechanisms, including prevention of excitotoxicity, reducing the inflammatory response, fending off oxidative stress, and stabilizing mitochondrial activity, these ions play a crucial central role in the regulation of the pathological process and eventual repair of nervous system injury, as depicted in **Figure 2**. As a result, controlling magnesium ion levels or administration of magnesium ion supplements has emerged as a very potential therapeutic intervention target.

## Safety of Magnesium

The magnesium ion is a crucial divalent cation in the body. The overall quantity of magnesium in healthy adults is approximately 20–28 g, of which 50%–60% is stored in bones, 34%–39% in muscles, soft tissues, and organs, and merely 1%–2% in blood and extracellular fluid. The magnesium content in serum varies from around 0.7 to 1.0 mM (Fiorentini et al., 2021; Kröse and de Baaij, 2024). Additionally, serum magnesium is present in three forms: approximately 5%–15% is bound to anions such as phosphate, citrate, or bicarbonate; 20%–30% is bound to proteins; and approximately 55%–70% exists in free form, which is regarded as the primary biologically active form of magnesium. Magnesium ions in the human body are primarily absorbed by the intestine, particularly through the paracellular pathway in the small intestine and the transcellular pathway in the colon (Schweigel and Martens, 2000).

Magnesium exhibits high biocompatibility, indicating that it does not elicit harmful immune responses or adverse reactions in the body and thus rendering it safe for medical applications (Seetharaman et al., 2023). Unlike permanent implants made of materials such as titanium or stainless steel, which tend to remain in the body for long periods without being affected (Hayes and Richards, 2010), magnesium can degrade naturally in the body by breaking down into harmless magnesium ions, which can be metabolized and excreted (Bairagi and Mandal, 2022). This helps minimize the need for surgical removal of the implant after its purpose has been served, thereby minimizing the risk of problems and overall treatment expenses. In addition to its fascinating biological features, magnesium possesses a unique combination of mechanical properties similar to those of natural bone (Staiger et al., 2006). Consequently, magnesium-based materials can be employed as temporary implants for orthopedic injuries.

Magnesium-based compounds were first utilized within the human body over a century ago. The history of magnesium dates back to 1808, when Sir Humphry Davy first discovered the element magnesium. Shortly thereafter, in 1833, his assistant Michael Faraday produced magnesium metal by electrolyzing anhydrous magnesium chloride (Witte, 2010). Subsequently, people began considering the clinical application of magnesium, with Dr. Edward C. Huse using magnesium wire as a ligature for hemostasis in 1878 (Huse, 1878). More recently, in 1892, Dr. Erwin Payr of Graz, Austria, conducted the first experiments on magnesium degradation and applied high-purity magnesium plates to the knee joints of dogs and rabbits, hoping to restore joint stability and motor function. This led to a deeper understanding of magnesium metal. Consequently, in 1900, Dr. Payr used magnesium tubes in cardiovascular applications as connectors for vascular anastomosis. He also used magnesium tubes for sutures in nerve repair. In 1906, Lambert was the first to use magnesium-based materials to treat clinical fractures. These findings represent the various clinical applications of magnesium metal. Nevertheless, all of these attempts ultimately failed due to the quick corrosion of magnesium in the body’s hostile environment. Subsequently, to address this issue, magnesium alloys, magnesium-based MGs, and surface-modified magnesium materials were developed (Zhou et al., 2024).

From a physiological standpoint, magnesium is an optimal implant material from the perspective of biocompatibility alone. However, the corrosion product of magnesium consists of magnesium ions (Song and Atrens, 1999) as well as hydrogen, which is a major corrosion byproduct of magnesium. When inserted into the body, a magnesium implant will corrode swiftly in the body’s hostile environment, generating many bubbles. Furthermore, the accelerated corrosion of magnesium generates a substantial quantity of hydrogen within a day, impeding the healing of the surgical site, inducing tissue necrosis, and resulting in patient pain. Moreover, corrosion of magnesium metal produces OH^–^, resulting in the alkalinization of bodily fluids. Consequently, implantation of magnesium components in the human body causes local elevation of the pH levels of the surrounding bodily fluids, substantially disrupting the equilibrium of physiological processes and harming human health. Conversely, magnesium supplementation for neurological disorders may result in hypermagnesemia (Verma and Ogata, 2023).

## Present Status of Clinical Research

A search of trials on the use of magnesium in neurological diseases in ClinicalTrials.gov identified 258 trials encompassing conditions such as migraine, Alzheimer’s disease, Parkinson disease, traumatic brain injury, and peripheral neuropathy. These quintessential clinical studies demonstrated that magnesium supplementation positively influences the treatment of central nervous system disorders (**Additional Table 3**).

**Additional Table 3:** Examples of neurological disorders treated with magnesium in ClinicalTrials.gov

Study title	NCT identifier	Recruitment status	Enrollment (*n*)	Study design	Start date	Interventions	Primary outcomes	Adverse events	Sponsor/collaborator
Magnesium versus prochlorperazine versus metoclopramide for migraines (MAGraine)	NCT05967442	Completed	157	Interventional studies	October 1, 2024	Intravenous administration of magnesium sulfate compared with intravenous injections of metoclopramide and prochlorperazine.	Exhibits magnesium tolerance, possesses favorable safety profile, and may be efficacious in migraine treatment.	Flushing is the most frequent major adverse event and is self-relieving.	Wake Forest University Health Sciences
Magnesium L-threonate for the enhancement of learning and memory in people with dementia	NCT02210286	Completed	17	Interventional studies	February 2, 2021	Vitamins C and D combined with L-threonate administered orally.	The use of L-magnesium threonate may improve learning and memory in individuals with mild to moderate dementia.	Not specified	Stanford University
MLD10 in the prevention of migraine in adults	NCT02322333	Completed	157	Interventional studies	October 9, 2020	Administer two tablets bi-daily. MLD10.	Oral MLD10 can diminish the duration and intensity of migraines.	Not specified	Pharmalyte Solutions LLC
Acetaminophen and Ibuprofen with and without magnesium and primary migraine in childhood	NCT01756209	Completed	160	Interventional studies	March 25, 2020	Acetaminophen and ibuprofen, both with and without magnesium.	Magnesium can diminish the duration of pain.	Not specified	University of Catanzaro
Mild hypothermia and supplemental magnesium sulfate infusion in severe traumatic brain injury (TBI) subjects	NCT01333488	Completed	6	Interventional studies	September 30, 2015	Low temperature + magnesium sulfate injection.	The incorporation of magnesium sulfate may facilitate the recovery from traumatic brain injury.	No significant adverse reactions observed.	United States Department of Defense
Calcium and magnesium in preventing peripheral neuropathy caused by ixabepilone in patients with breast cancer	NCT00998738	Completed	1	Interventional studies	February 4, 2016	Give magnesium sulfate and calcium gluconate.	Calcium given with magnesium may prevent or slow the development of peripheral neuropathy.	Due to the accumulation of only one patient, confidentiality constraints inhibit the reporting of the patient's information.	Mayo Clinic

This table summarizes classic cases of nerve injury repair using magnesium-based materials, as documented in ClinicalTrial.gov, providing evidence for future use of magnesium for treatment of neurological disordes. It also shows the efficacy of magnesium supplements in treating central nervous system diseases. MLD10: Dehydrate magnesium L-lactate; TBI: traumatic brain injury.

## Limitations

This article examines the beneficial effects and applications of magnesium-based materials and magnesium ions in SCI, PNI, brain injury, and optic nerve injury.

For the treatment of SCI, Wiseman et al. (2009) constructed too few experimental models, potentially yielding inaccurate results in comparison with steroid treatment. The study by Kaptanoglu et al. (2003b) did not include human experiments, highlighting the need for further research. Moreover, while Sperl et al. (2019) conducted a prospective study on humans, the population size was too small and the results were not representative. Therefore, much research is needed on the role of magnesium in the treatment of SCI.

For the treatment of PNI, Hopkins et al. (2017) proved that magnesium wire shows some effectiveness in short-gap nerve injury, but does not show good regeneration ability in long-gap injury. Similar to the findings obtained by Hopkins et al. (2017), Vennemeyer et al. (2015) also found that magnesium wire is not suitable for long-gap injury. Bhat et al. (2024) conducted *in vitro* experiments for the treatment of PNI by magnesium alloy. However, the *in vivo* environment is more complex and dynamic than the *in vitro* environment, and the potential differences in corrosion rates between the two environments can yield different results. Therefore, further exploration is needed. To solve the problem of the degradation rate of magnesium alloy, Almansoori et al. (2018) modified the surface of the magnesium alloy with HA, but the surface of the catheter was too rough and not suitable for cell adhesion. Therefore, although this experiment proved that a modified magnesium-based material promotes nerve repair, the modified materials require further exploration. In addition, magnesium-based MG can also solve the degradation problem of magnesium metal, but no team has studied the direct use of MG for the repair of PNI. In addition, magnesium-based MG is in its infancy and is difficult to produce, and sufficiently large samples are still difficult to obtain.

Although magnesium ions have beneficial effects on the repair of optic nerve and brain damage, a thorough understanding of the types of magnesium ions that show such effects and their mechanisms of action is lacking because of the limited literature on this topic.

In conclusion, literature on the application of magnesium ions and magnesium-based products in the treatment of the aforementioned nerve injuries is lacking. Therefore, although magnesium ions and various functionalized materials derived from this element have shown great potential in the treatment of nervous system injury, their clinical application is limited.

## Conclusion and Outlook

This study provides an overview of the mechanisms underlying nervous system injury, including excitotoxicity, mitochondrial dysfunction, neuroinflammation, and oxidative stress after nerve damage. It also investigates the functions of magnesium ions in neuron repair by lowering oxidative stress, protecting mitochondria, minimizing excitotoxicity, and exerting anti-inflammatory activity. It also covers the use of metallic magnesium, magnesium alloys, surface-modified magnesium-based materials, and magnesium-based MG to facilitate restoration of the nervous system. Nervous system injuries, such as those to the brain, spinal cord, peripheral nerves, or optic nerves, show both commonalities and differences. Nonetheless, the previously reported functions of magnesium ions in nervous system traumas have favorable effects on healing procedures.

While magnesium-based biomaterials have shown great promise for use in nerve repair. However, the use of magnesium-based biomaterials for nerve repair is currently associated with several limitations. Research on the use of magnesium ions and magnesium-based materials to treat injuries, including those involving the brain, spinal cord, optic nerve, and peripheral nerve, is currently limited. Consequently, future efforts should prioritize the investigation and therapeutic utilization of magnesium ions and magnesium-based materials in the therapy of the aforementioned nerve injuries.

In summary, the field of magnesium-based biomaterials still offers great potential for nerve repair with numerous opportunities and challenges. The advancement of research and the subsequent application in clinical practice will require researchers’ collaborative involvement and efforts to ultimately benefit patients.

## Additional files:

***Additional Table 1:*** *Various magnesium-based materials used for repair of peripheral nerve injury.*

***Additional Table 2:*** *Effects of magnesium ions.*

***Additional Table 3:*** *Examples of neurological disorders treated with magnesium in ClinicalTrials.gov.*

## Data Availability

*All relevant data are within the paper and its Additional files.*
